# An Extranodal Site of Diffuse Large B-cell Lymphoma Presenting as Ovarian Cancer

**DOI:** 10.7759/cureus.34337

**Published:** 2023-01-29

**Authors:** Laila Jaouani, Adil Zaimi, Ouissam Al Jarroudi, Soufiane Berhili, Sami Aziz Brahmi, Said Afqir

**Affiliations:** 1 Faculty of Medicine and Pharmacy, Mohammed First University, Oujda, MAR; 2 Medical Oncology, Hassan II Oncology Centre, Mohammed VI University Hospital, Oujda, MAR; 3 Medical Oncology, Faculty of Medicine and Pharmacy, Mohammed First University, Oujda, MAR; 4 Medical Oncology, Mohammed VI University Hospital, Oujda, MAR; 5 Radiation Oncology, Faculty of Medicine and Pharmacy, Mohammed First University, Oujda, MAR; 6 Oncology, Faculty of Medicine and Pharmacy, Mohammed First University, Oujda, MAR

**Keywords:** r-chop-rituximab, cd20+, immunohistochemical panel, exploratory laporotomy, ovarian lymphoma

## Abstract

Due to its uncommon nature, primary ovarian lymphoma has no clinical particularities and can be confused with other ovarian cancers. It poses a twofold diagnostic and therapeutic challenge. An anatomopathological and immunohistochemical study is the crucial step in the diagnosis. Our case was a 55-year-old woman diagnosed with an Ann Arbor stage II E ovarian non-Hodgkin's lymphoma who initially presented with a painful pelvic mass. This case reflects the major role of an immunohistochemical study in the diagnosis workup, leading to the appropriate management of such rare tumors.

## Introduction

Primary ovarian lymphoma remains an extremely rare location, forming 1.5% of all ovarian tumors [1]. It can be primary or secondary, unilateral or bilateral. Lymphoma is a disease that usually involves lymphoid and hematopoietic tissues, but any organ can be affected [2]. Several hypotheses have been formulated to explain the pathophysiology of lymphomatous affection of the ovary. Some authors suggest a local reaction of systemic disease [3,4] while others are strongly convinced by the primary character [5-7].

Clinical and imaging findings are not specific, and the diagnosis of primary ovarian lymphoma is often obtained after surgery, which is not the standard of care.

In the published literature, there has been a wide range of publications from 1946 to the present day, from a few reported cases to case series [8-10]. Our case aims to add to the previous data on this topic.

## Case presentation

**Figure 1:**
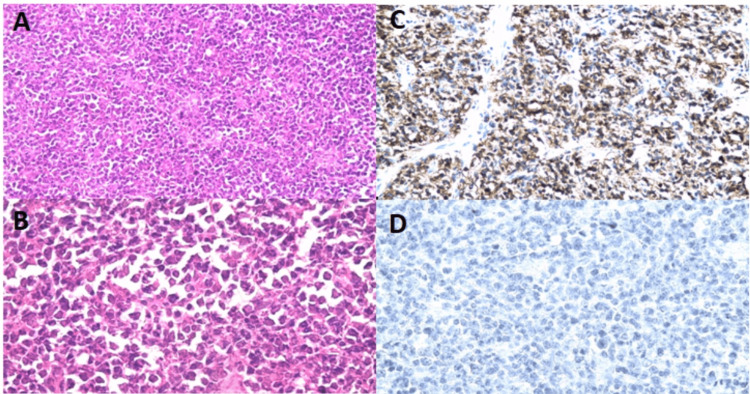
Germinal center B-cell type diffuse large B-cell ovarian lymphoma A- Diffuse non-cohesive round cell tumor proliferation (HE; 200×) B- Details of the ovary tissues under the microscope. In tumor tissues (Image A) (HE; 400×) C- Intense positive immunostaining of tumoral cells for CD 20 (200×) D- No tumor cells express inhibin-alpha marker (400×) HE: Hematoxylin and eosin stain

The patient was 55 years old, menopausal, with no previous pathological history. She was consulted for pelvic pain of the pressure type with lumbar irradiation without any other gynecological or extra-gynecological signs. She had a pelvic ultrasound that showed a suspected hypoechoic right ovarian mass of 5 cm x 6 cm. Tumor markers were cancer antigen 125 (CA125), B human chorionic gonadotropin (BHCG), alpha-fetoprotein (AFP), and carcinoembryonic antigen (CAE) were normal. Given that ovarian carcinoma was initially suspected, an exploratory laparotomy with optimal excisional surgery (total hysterectomy with bilateral adnexectomy, omentectomy, appendectomy, and peritoneal biopsies) was performed. Surgical exploration revealed a right ovarian mass measuring 8x6x5 cm, adjacent to a tube. Anatomopathological analysis revealed an undifferentiated right ovarian process that could correspond to an adult granulosa tumor, the left ovary free of tumor infiltration, a fibro-myomatous uterus, and chronic appendicitis. No peritoneal involvement was found. The immunohistochemical study (Table 1) confirmed the diagnosis of a diffuse, ovarian, large B-cell non-germ-center lymphoma (Figure 1).

**Table 1:** A summary of the immunostaining findings panel

Antibodies used	Staining
Anti-CD45	Positive
Anti-CD20	Positive
Anti-KI67	Positive (estimated at 40%)
Anti-CD3	Negative
Anti-BCL2	Negative
Anti-CD10	Negative
Anti-BCL6	Negative
Anti-CD23	Negative
Anti-Inhibine alpha	Negative
Anti-CK	Negative

A postoperative thoracoabdominal-pelvic CT scan was reported to be without abnormality. On admission, the patient was PS=1, hemodynamically and respiratorily stable and the somatic examination found a transverse abdominal scar. The rest of the clinical examination was unremarkable. A pre-therapeutic workup was requested, including a bone marrow biopsy that showed a moderately rich marrow, polymorphic, without any territory suspected of malignancy. A high lactate dehydrogenase level of 286 IU/l, an elevated sedimentation rate of 41 mm, 34 mm at H1 and H2, and viral serologies for hepatitis B, C, and HIV were all reported as negative. The patient received six courses of R-CHOP every 21 days (intravenous Rituximab 375 mg/m2; Cyclophosphamide iv 750 mg/m2; Doxorubicin iv 50 mg/m2; Vincristine 1.4 mg/m2; Oral Prednisone 40 mg/m2 1-5 days) with complete remission. Normalization of lactate dehydrogenase to 187 and sedimentation rate to 9 mm in the first hour and 15 mm in the second hour were noted. A follow-up CT scan was performed without abnormality.

The patient was put under surveillance. She is still alive without any incident 36 months after the end of chemotherapy.

## Discussion

Primary ovarian involvement by malignant non-Hodgkin's lymphoma is an unusual and rarely reported form. It constitutes 0.5% of all non-Hodgkin's lymphomas [1].

Ever since Walther in 1934, numerous physiopathological hypotheses have been put forward to explain the primitive nature of the disease. According to Woodruff, the occasional presence of lymphocytes disseminated at the level of the cortex and rarely at the level of the ovarian stroma and follicles are at the origin of the genesis of lymphomatous damage at this level [11]. Subsequently, Durfee and Oberling in 1937 suggested that although the ovary is not a lymphoid organ, the lymphoid infiltrate may be found accidentally and especially in reaction to chronic inflammation in pelvic pathologies of infectious origin, autoimmune ovaritis, and sometimes even in the case of a pre-existing teratoma [7]. In 1976, Fox and Langley proposed three well-defined criteria for the diagnosis of primary ovarian lymphoma.

First criterion: At the moment of diagnosis, the lymphoma is clinically confined to the ovary and investigation reveals no lymphomatous localization elsewhere. However, ovarian lymphoma is considered primary even if there is adjacent lymph node involvement or neighborhood structures.

Second criterion: The pre-treatment evaluation, including the blood count, blood smear, and bone marrow biopsy, must be normal.

Third criterion: The time interval between ovarian lymphoma and extra-ovarian metastatic relapse should be within several months [12].

Our case meets all three Langley criteria.

Primary ovarian non-Hodgkin's lymphoma of the diffuse large cell type is the histological subtype most often incriminated in gynecological tract involvement following Burkitt's lymphoma [13]. Usually, the involvement is not always unilateral because the bilateral form has been frequently reported by authors, notably in seven case series with 41-71% [9,14-16]. Our patient had a unilateral right ovarian lymphoma. As with most cancers, age is an important risk factor. This is not the case for primary ovarian non-Hodgkin's lymphoma. In most publications, the median age of patients was 42 to 47 years [17,18], but it can occur at any age. Senol et al. reported a median age of 57 years. Our patient was 50 years old.

The clinical picture includes non-specific signs. Most patients arrived with the well-known triad of abdominal-pelvic pain, abdominal-pelvic mass, and peritoneal effusion. Other signs that have been reported include weight loss, fever, and sometimes metrorrhagia [9,14-15]. A clinical examination may find hepatosplenomegaly or palpable adenopathy [17]. In our case, the patient was diagnosed with isolated pelvic pain.

Some blood tests have diagnostic and even prognostic values. Elevated CA125 and lactate dehydrogenase levels are often correlated with advanced disease, poor therapeutic response, and poor survival [19-22]. In the series of Zidane et al., 45% of patients with an elevated CA125 level had an estimated five-year overall survival of 50% for low-grade lymphoma and 27% for the aggressive form [19]. In our case, the CA125 level was normal at 17.8 IU/ml and the lactate dehydrogenase was elevated at 286 IU/ml.

Although there is no typical imaging of ovarian lymphoma, the radiological workup has an important place in characterizing lymphomatous involvement. Pelvic ultrasound is the first test to be requested to explore a pelvic mass. In the majority of published cases, the ultrasound appearance of the lesions was aspecific. It presented as a homogeneous hypoechoic solid mass with slight vascularization on Doppler [18]. This was the case with our patient.

The CT scan most often reveals a well-limited, hypodense tissue mass with mild enhancement upon contrast injection. Magnetic resonance imaging shows homogeneous masses with a moderately hypointense appearance in T1 and slightly hyperintense in T2, with moderate enhancement after gadolinium injection [18,23].

Ovarian lymphoma cannot be diagnosed preoperatively. The extemporaneous examination of this tumor is difficult, and the lymphoma is often diagnosed as an undifferentiated carcinoma [24]. The histological differential diagnosis may be an epithelial tumor, a dysgerminoma, a granulosa cell tumor, or even a teratoma [25].

It is the immunohistochemical study that establishes the definitive diagnosis. According to European guidelines, a diagnosis of diffuse large B cell lymphoma should be confirmed by immunophenotypic testing, either by immunohistochemistry (IHC), or flow cytometry, or a combination of both techniques. The immunohistochemical panel includes CD20, CD79a, BCL6, CD10, MYC, BCL2, Ki67, IRF4, CyclinD1, CD5, and CD23 [26]. The most commonly published subtype in the literature is the diffuse large-cell B-cell non-Hodgkin's lymphoma, which is characterized by high expression of CD20, CD45, and KI 67 [27]. In our case, there was positivity for CD45, CD20, and KI67 of 40% and negativity for BCL2, CD10, BCL6, CD23, inhibin alfa, and cytokeratin.

After the diagnosis has been confirmed, a thoracic-abdominal-pelvic CT scan is used to stage the disease. An osteomedullary biopsy is also used for this purpose. A positron emission tomography (PET) scan is indicated for staging, treatment evaluation, and detection of recurrences [7,28].

Our patient underwent a postoperative thoracic-abdominal-pelvic CT scan, which was normal. Similarly, the osteomedullary biopsy did not reveal any infiltration.

The therapeutic management of non-Hodgkin's lymphoma with an ovarian origin requires a multidisciplinary consultation meeting and the treatment is decided according to the histological subtype.

The choice of treatment depends initially on the patient's age, general condition, cardiac status, and stage of the disease. Before starting treatment, the risks of infertility and the possibilities of fertility preservation must be discussed, depending on the type of treatment proposed. Treatment is based on systemic chemotherapy with Cyclophosphamide, Doxorubicin hydrochloride, Vincristine (Oncovin), and Prednisone) associated with an anti-CD 20 monoclonal antibody (Rituximab) for a total of six cycles every 21 days [28]. Our patient received six cycles of adjuvant R-CHOP with complete remission.

## Conclusions

Any ovarian mass is not always an epithelial tumor, hence the interest in an exploratory laparotomy to avoid unnecessary radical surgery. A histological study with immunohistochemistry remains the cornerstone for making the diagnosis and guiding the therapeutic treatment.
